# Hybrid Closed Wedge High Tibial Osteotomy Maintains the Leg Length After Surgery Compared With Open Wedge High Tibial Osteotomy

**DOI:** 10.7759/cureus.57953

**Published:** 2024-04-10

**Authors:** Shu Takagawa, Naomi Kobayashi, Yohei Yukizawa, Kunihito Hirotomi, Shota Higashihira, Yutaka Inaba

**Affiliations:** 1 Orthopaedics Surgery, Yokohama City University Medical Center, Yokohama, JPN; 2 Orthopaedic Surgery, Yokohama City University Medical Center, Yokohama, JPN

**Keywords:** open wedge osteotomy, closed wedge osteotomy, knee osteoarthritis/ koa, leg length, osteotomy knee

## Abstract

Background: This study aimed to assess the changes in leg length following open wedge high tibial osteotomy (OWHTO) and hybrid closed wedge high tibial osteotomy (h-CWHTO) and whether the change in leg length was associated with preoperative radiographic factors and the change in planned opening or closing width.

Methods: We retrospectively evaluated the data of patients who underwent OWHTO (n=57) and h-CWHTO (n=31) between 2016 and 2019. Standing full-length anteroposterior radiographs were obtained preoperatively and one year postoperatively. Changes in the lower leg and tibial length were measured using radiography, and the planned opening or closing width was decided via operative planning. Flexion contracture was examined preoperatively and one year postoperatively using a goniometer, and the correlation factors and changes in leg length were analyzed using Spearman’s rank correlation.

Results: In the OWHTO group, the lower leg was significantly longer by a mean of 6.0±8.7 mm compared to that preoperatively (p<0.01); however, no significant difference was observed in the h-CWHTO group (mean, -0.56±11.6 mm) (p=0.788). In the OWHTO group, flexion contracture did not improve after surgery, however, in the h-CWHTO group, flexion contracture significantly improved from -7.1±7.0 degrees to -4.7±6.2 degrees postoperatively (p<0.01). No radiographic factors or bone opening or closing width were associated with changes in leg length in both groups.

Conclusion: OWHTO led to a significant elongation of the lower leg while leg length was maintained post-h-CWHTO. However, the changes in leg length following both OWHTO and h-CWHTO were not predictable from preoperative radiographic factors or changes in bone width.

## Introduction

Medial compartment osteoarthritis (OA) of the knee is the most common degenerative joint disorder in the elderly [1]. In Japan, knee OA affects more than 40% of men and more than 60% of women over the age of 40 years [2]. Around-knee osteotomy, including high tibial osteotomy (HTO), is indicated for patients with young and high-activity patients with knee osteoarthritis that affects only one compartment [3]. HTO is mainly categorized as either closed wedge HTO (CWHTO) [4] or open wedge HTO (OWHTO) [4].

Hybrid CWHTO (h-CWHTO), developed by Takeuchi et al., is an improved surgical technique compared with conventional CWHTO that can help reduce the amount of bone block removed. Several studies have reported leg length discrepancies after surgery between OWHTO and CWHTO [5-8]. Theoretically, h-CWHTO is superior to conventional CWHTO in terms of leg length retention after surgery. Leg length change after HTO affects the gait and induces musculoskeletal disorders, however, this phenomenon might not be fully recognized by orthopaedic surgeons [9,10]. Consequently, it is crucial to develop methods to predict these changes before performing surgery. A recent study reported that using digital planning software for HTO, such as mediCAD® Classic, enabled surgeons to simulate the degree of change in leg length post-surgery [11]. However, h-CWHTO is a three-dimensional osteotomy in which bone fragments are separated; consequently, changes in leg length do not always conform to the expectations of two-dimensional planning. Therefore, measurements of actual radiographic parameters and assessment of potential determinants or correlates of changes in leg length following surgery are meaningful. This study sought to answer the question: Does h-CWHTO help preserve the leg length after surgery compared with OWHTO?

Therefore, the purpose of this study was to assess the change in leg length following OWHTO or h-CWHTO in patients with OA. We further examined the association of changes in leg length with preoperative radiographic parameters and the planned opening or closing of the osteotomy gap. This article was previously posted on the Research Square preprint server on August 16, 2022.

## Materials and methods

Study design

**Figure 1:**
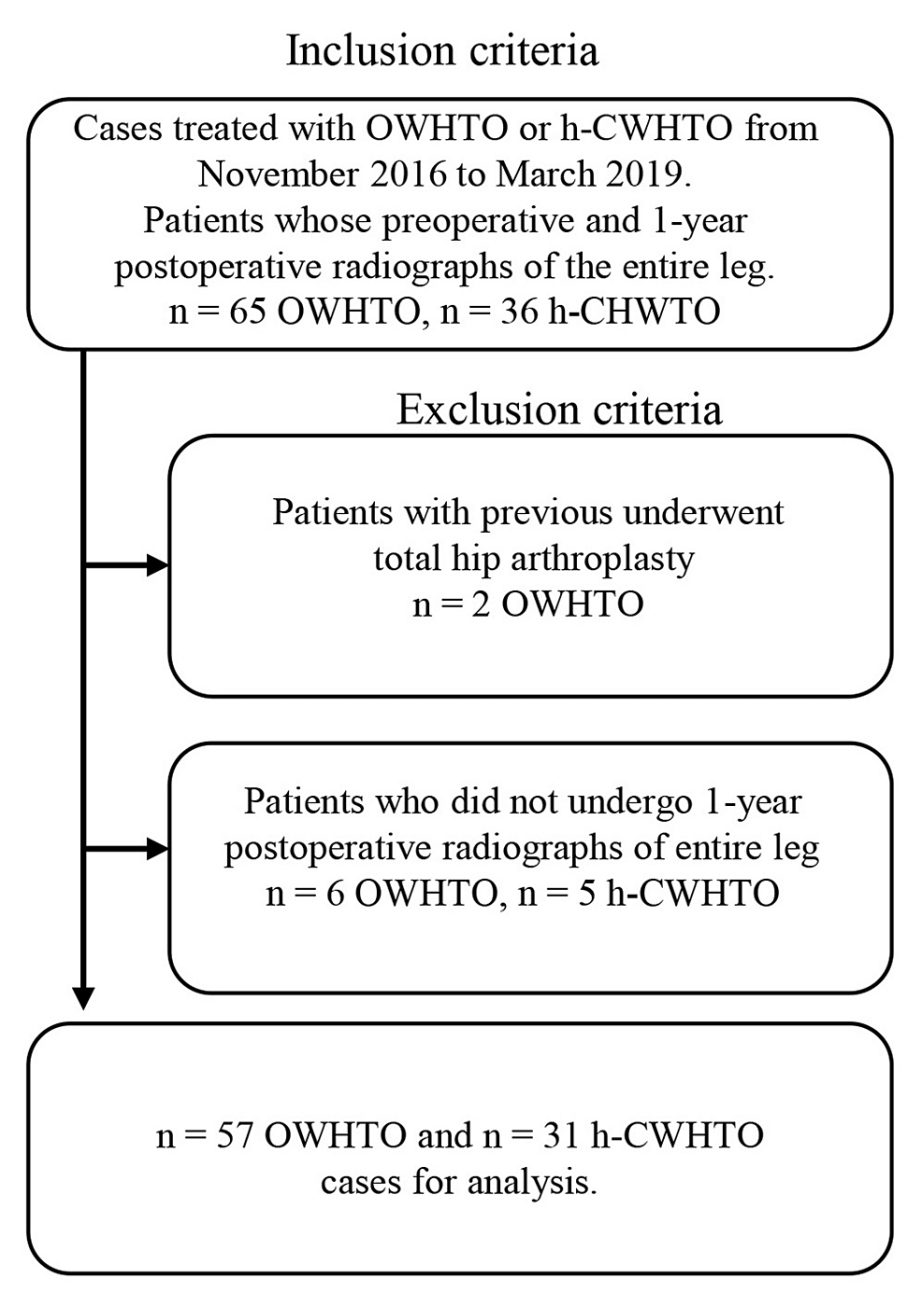
Study flow chart The figure depicts the inclusion and exclusion criteria of the study participants. OWHTO: opening wedge high tibial osteotomy; h-CWHTO: hybrid closed wedge high tibial osteotomy.

The study evaluated the data of patients who underwent OWHTO or h-CWHTO between November 2016 and March 2019 at Yokohama City University Medical Center, Japan. The inclusion criteria were as follows: OWHTO or h-CWHTO performed for medial compartment osteoarthritis. The exclusion criteria were as follows: (1) patients who previously underwent total hip arthroplasty, and (2) patients for whom one-year postoperative radiographs of the entire leg in the anteroposterior view in the standing position were not obtained (Figure 1).

OWHTO and h-CWHTO were performed by two experienced, senior orthopedic surgeons. The indications for both surgical treatments were medial compartment knee OA, alone or combined with patellofemoral OA, and impaired activity of daily living due to persistent knee pain after at least three months of conservative treatment. OWHTO was performed in cases with a correction angle of <15 degrees and flexion contracture of <15 degrees and without failure of the anterior cruciate ligament [12]. h-CWHTO was performed in cases where OWHTO was contraindicated, and flexion contracture was <20 degrees.

Preoperative planning

**Figure 2:**
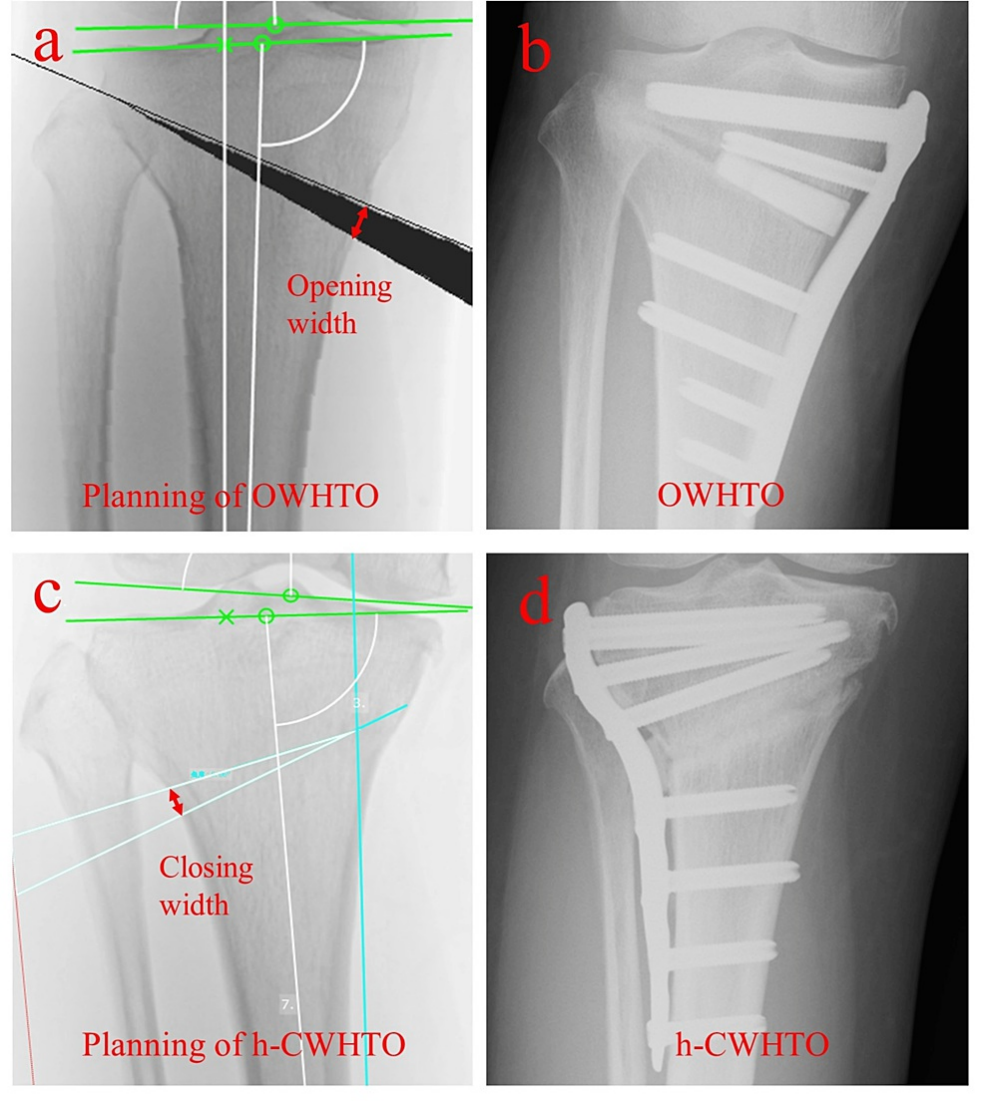
Preoperative planning of OWHTO and h-CWHTO (a) Planning of OWHTO; (b) Postoperative radiograph of the right knee after OWHTO; (c) Planning of h-CWHTO; (d) Postoperative radiograph of the right knee after h-CWHTO OWHTO: Opening wedge high tibial osteotomy; h-CWHTO: hybrid closed wedge high tibial osteotomy.

Radiographic measurements of the entire leg in the anteroposterior view in the standing position were used. Both surgical procedures were aimed at achieving a mechanical axis, from the center of the hip to the center of the ankle, that passes 62% laterally from the medial edge of the tibial plateau. The preoperative weight-bearing line (WBL) was defined by drawing a line from the center of the femoral head to the midpoint of the proximal talar joint surface. A new WBL was drawn from the center of the femoral head, passing 62% laterally from the medial edge of the tibial plateau to the level of the ankle joint line. [13] In the OWHTO group, the hinge point of the osteotomy was defined as a point 5 mm medial to the lateral cortex on the first cut line of the tibia on the osteotomy line. In the h-CWHTO group, the hinge point was set at an approximately 2:1 ratio from the medial border of the first cut line of the tibia on the proximal osteotomy line (Figure 2).

Surgical technique and postoperative rehabilitation

The surgical procedures for OWHTO and h-CWHTO were performed as previously described by Takeuchi et al. [14,15]. Briefly, arthroscopy was routinely performed before osteotomy to evaluate the cartilage, meniscus, and ligaments for both procedures. In the OWHTO group, the medial proximal tibia was exposed using an oblique incision, and the superficial fibers of the medial collateral ligament were distally released. Two Kirschner wires, 35 mm distal to the medial proximal tibial joint surface to the tibiofibular joint, were used as place markers for the saw cut. A separate ascending cut for the biplanar osteotomy was made 1.5 cm behind the tibial tuberosity in the frontal plane, at an angle of 100-110 degrees from the first osteotomy plane. The osteotomy site was opened until the desired alignment was reached. The gap created by the OWHTO was filled with β-tricalcium phosphate (Olympus Terumo Biomaterials Corp., Tokyo, Japan); the amount used was determined during preoperative planning. The locking plate was fixed using eight locking screws.

In the h-CWHTO group, fibular osteotomy was performed prior to tibial osteotomy, and segmental resection was performed to remove an approximately 10-20 mm length of bone, enabling tibial correction through a separate incision. A longitudinal, lateral skin incision was made, first on the anterior aspect of the knee and then on the lateral side of the tuberosity. Two Kirschner wires were inserted, from 35 mm distal to the lateral proximal tibial joint surface to 15 mm distal to the medial proximal tibial joint surface. The hinge point divided the proximal tibial osteotomy line in an approximately 2:1 ratio; a Kirschner wire was percutaneously inserted toward the hinge point in the anterior-to-posterior direction. To determine the distal osteotomy line, a dedicated CWHTO goniometer (Mizuho Medical, Tokyo, Japan) was set at the Kirschner wire that was inserted at the hinge point; two Kirschner wires were then inserted from this point to the hinge point. The wedge angle, which is equal to the correction angle, was determined through preoperative planning. The wedge between the first and second Kirschner wires was carefully removed using a bone saw and chisel. For the biplanar osteotomy, a separate, ascending cut was subsequently made behind the patella tendon insertion in the frontal plane, retaining the tibial tuberosity at a thickness of approximately 10 mm. Finally, the medial cortex was cut according to the first osteotomy line. Once the two bone fragments were compressed until the desired alignment was reached, a locking plate was used to fix the osteotomy site.

In the OWHTO group, patients were able to attempt full weight-bearing using support equipment the day after surgery. In the h-CWHTO group, patients were able to attempt 50% partial weight-bearing using support equipment seven days postoperatively; full weight-bearing with a T-cane was permitted after two weeks. In the OWHTO group, the opening gap was defined as the elevation of wedge-shaped β-tricalcium phosphate created by planned opening width. In the h-CWHTO group, the closing gap was defined as the height of the resected bone fragment.

Clinical evaluation

Patient data were collected, including demographics, age, height, weight, body mass index (BMI), and occurrence and time intervals of subsequent contralateral knee arthroplasty. Flexion contracture was also evaluated pre- and one-year postoperatively using a goniometer.

Radiological evaluation

**Figure 3:**
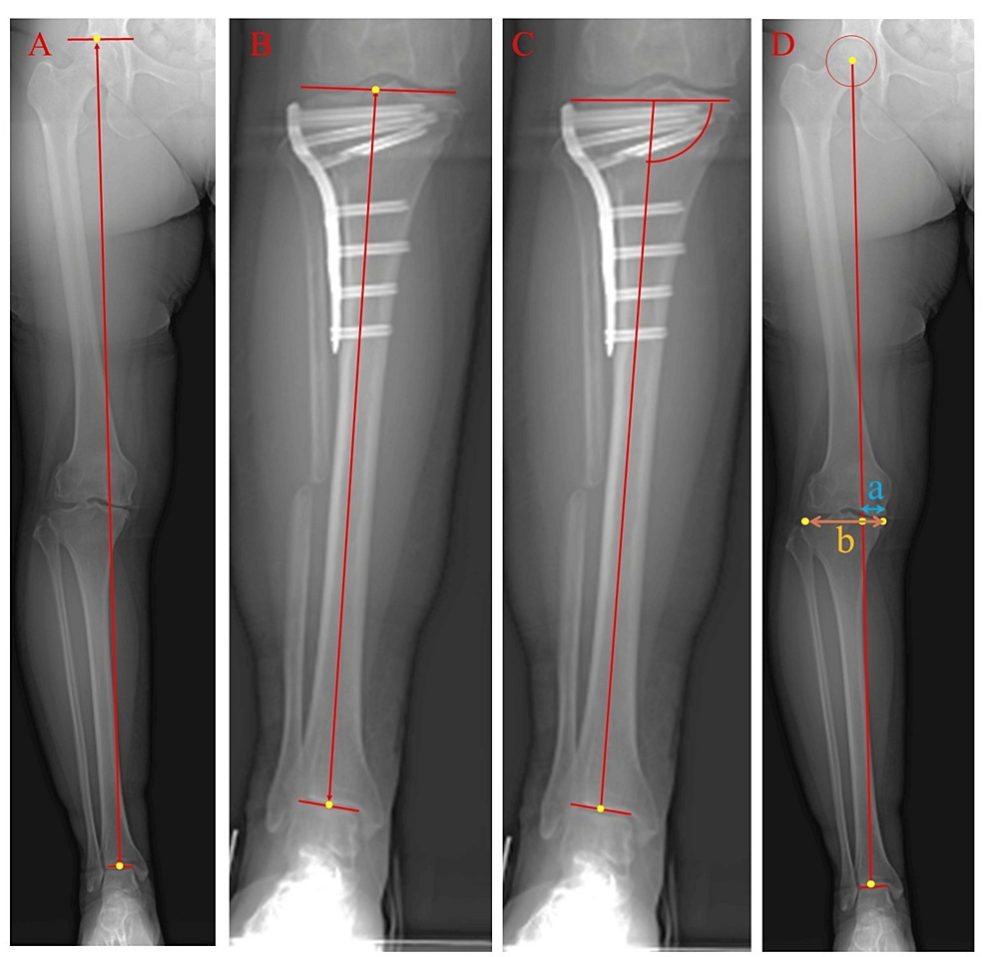
Measurements of lower leg parameters on single-leg-stance anteroposterior radiographs (a) The length of the lower leg was defined as the length of the line from the top of the femoral head to the center of the midpoint of the tibial talar joint; (b) The length of the tibia was defined as the length of the line from the midpoint of the tibial eminences to the center of the midpoint of the tibial talar joint; (c) The medial proximal tibial angle was defined as the angle between the tibial mechanical axis and the articular surface of the proximal tibia; (d) % Mechanical axis was calculated as follows: a/b × 100.

Anteroposterior full-length lower limb radiographs in the standing position were obtained preoperatively and one year postoperatively. Radiographs were projected using the Fuji Computed Radiography system, and various parameters were measured using Fujifilm OP-A software (Fujifilm Corporation, Tokyo, Japan). The lower leg and tibial length, medial proximal tibial angle (MPTA), and % mechanical axis (% MA) were measured (Figure 3).

Lower limb length was defined as the length of the line from the top of the femoral head to the center of the midpoint of the tibial talar joint. The tibial length was defined as the length of the line from the midpoint of the tibial eminence to the center of the midpoint of the tibial talar joint. MPTA was defined as the angle between the mechanical axis of the tibia and the tibial plateau. The % MA was calculated by measuring the distance from the medial edge of the proximal tibia to the point where the mechanical axis intersects the proximal tibia; this was then divided by the width of the proximal tibia. A change in lower limb length and tibial length was defined as postoperative lower leg length minus preoperative lower leg length. Additionally, the changes in lower limb length and tibial length were compared between the OWHTO and h-CWHTO groups.

Statistical analysis

Values are expressed as mean ± standard deviation (SD). Demographic data were analyzed using the Mann-Whitney U test and the Chi-square test. The comparisons between preoperative and postoperative parameters in the OWHTO and h-CWHTO groups were analyzed using the paired t-test for normally distributed data and Wilcoxon’s signed-rank test for non-normally distributed data, while the correlation factors and changes in leg length were analyzed using Spearman’s rank correlation coefficients. The amount of change in leg length and tibial length between the OWHTO and h-CWHTO groups was assessed using the Mann-Whitney U test. Statistical significance was set at P<0.05. All statistical analyses were performed using SPSS software (version 24.0; IBM Corp., Armonk, NY, USA).

## Results

In total, 57 cases of OWHTO and 31 of h-CWHTO were enrolled in the present study. The two groups showed no significant differences in age, sex, and height, while BMI was significantly higher in the h-CWHTO group (P=0.001). The clinical features of the patients enrolled in this study are summarized in Table 1.

**Table 1:** Patient demographics Values are presented as numbers or means ± standard deviations. aP-values were obtained using the Mann–Whitney U test. bP-values were obtained using the chi-square test. OWHTO: open wedge high tibial osteotomy; h-CWHTO: hybrid closed wedge high tibial osteotomy.

	OHWTO (57 cases)	h-CWHTO (31 cases)	P-value
Age	60.4±9.7	59.5±9.0	0.371^a^
Female/male	26/31	12/19	0.468^b^
Height, cm	159.0±9.6	159.8±9.1	0.724^a^
Body weight, kg	66.8±15.1	74.0±13.8	0.013^a^
Body mass index, kg/m^2^	26.3±5.0	28.8±4.1	0.001^a^

In the OWHTO group, flexion contracture did not improve after surgery; however, in the h-CWHTO group, flexion contracture significantly improved from -7.1±7.0 degrees before to -4.7±6.2 degrees after surgery. In the OWHTO group, lower leg length was significantly longer after surgery (mean: 6.0±8.7 mm); however, this significance was not observed in the h-CWHTO group (mean: -0.56±11.6 mm).

The tibial length was significantly longer after surgery by a mean of 5.3±6.4 cm in the OWHTO group; conversely, it was significantly shorter after surgery by a mean of -6.4±8.5 mm in the h-CWHTO group. MPTA and % MA significantly improved in both groups (Table 2).

**Table 2:** Summary of the parameters measured in the OWHTO and h-CWHTO group Values are presented as numbers or means ± standard deviations. a P-values were obtained using Wilcoxon’s signed-rank test. b P-values were obtained using the Mann–Whitney U test. MPTA: medial proximal tibial angle; % MA: % mechanical axis; OWHTO: open wedge high tibial osteotomy: h-CWHTO: hybrid closed wedge high tibial osteotomy.

	OWHTO			h-CWHTO		
	Preoperative	Postoperative	P-value	Preoperative	Postoperative	P-value
Flexion contracture (degree)	-3.7±4.7	-2.8±4.9	0.29^a^	-7.1±7.0	-4.7±6.2	0.002^a^
Lower leg length (mm)	753.5±48.8	759.5±48.3	0.0001^b^	759.5±51.6	758.9±50.5	0.7880^b^
Tibia length (mm)	336.4±23.8	341.7±23.0	0.026^b^	338.9±25.5	332.5±24.1	0.0001^b^
MPTA (degree)	85.4±1.8	93.4±2.3	0.0001^b^	84.9±1.8	93.6±3.0	0.0001^b^
% MA	20.3±12.8	65.0±7.6	0.0001^b^	6.4±15.2	62.0±15.8	0.0001^b^
Opening width (mm)	10.8±2.6	-	-	-	-	-
Closing width (mm)	-	-	-	10.3±2.2	-	-

**Figure 4:**
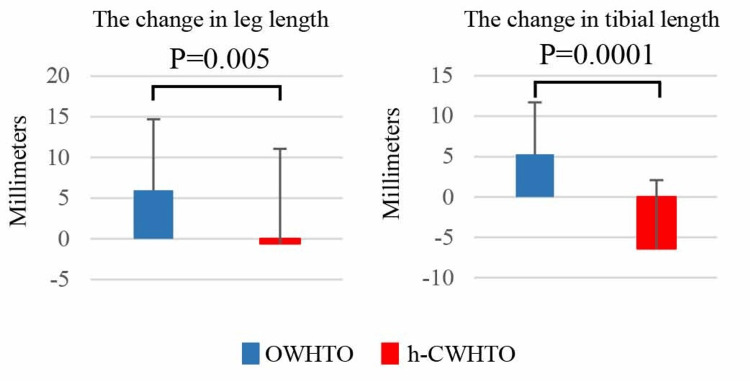
Changes in leg and tibial length Mean values are shown for open wedge high tibial osteotomy (OWHTO; blue bar), and hybrid closed wedge high tibial osteotomy (h-CWHTO; red bar). OWHTO: Opening wedge high tibial osteotomy; h-CWHTO: hybrid closed wedge high tibial osteotomy.

The amounts of change in leg length and the change in tibial length between the OWHTO and h-CWHTO groups were significantly different (Figure 4).

In either group, no significant correlations were observed between lower leg changes or parameters-including opening or closing width, preoperative flexion contracture, MPTA, and % MA (Table 3).

**Table 3:** Correlation between changes in lower leg length and parameters in the OWHTO and h-CWHTO group The P-values were obtained using Spearman’s rank correlation coefficients. MPTA: medial proximal tibial angle; % MA: % mechanical axis; OWHTO: open wedge high tibial osteotomy; h-CWHTO: hybrid closed wedge high tibial osteotomy.

	OWHTO				h-CWHTO			
	Opening width	Preoperative flexion contracture	Preoperative MPTA	Preoperative %MA	Closing width	Preoperative flexion contracture	Preoperative MPTA	Preoperative %MA
Change in lower leg length	Rho=0.245 P=0.08	Rho=0.225 P=0.109	Rho=-0.177 P=0.209	Rho=-0.025 P=0.109	Rho=-0.123 P=0.508	Rho=-0.031 P=0.869	Rho=0.219 P=0.319	Rho=-0.184 P=0.109

## Discussion

This study evaluated the change in leg length in OWHTO and h-CWHTO. The tibial length was significantly longer following OWHTO but was significantly shorter following h-CWHTO, and the lower limb length was significantly longer following OWHTO but was preserved after h-CWHTO. This phenomenon is thought to be due to the result that the severe varus is corrected to slight valgus alignment and flexion contracture was significantly decreased in the h-CWHTO group by decreasing posterior tibial slope. Predictive factors for changes in lower limb length were not identified in either group. Our findings suggest that h-CWHTO may be a feasible surgical procedure for patients not seeking leg length adjustment, although it can be challenging to predict changes in leg length discrepancy before surgery. 

In the present study, a 6.0-mm average increase in leg length was observed in the OWHTO group, while an average decrease of 0.56 mm, which was not statistically significant, was observed in the h-CWHTO group. These results were similar to those reported for other OWHTO procedures [5,8], which demonstrated increased leg lengths after surgery. In conventional CWHTO, leg length decreased by 5.7 mm in the study by Nerhus et al. [5] and by 2.7 mm in the study by Magnussen et al. [8]; both leg length changes were significantly different. This indicates that h-CWHTO was able to maintain lower leg length after surgery, unlike conventional CWHTO.

Compared with conventional CWHTO, h-CWHTO requires a small osteotomy gap and hinge point, which forms the center of rotation at the center of the tibia [15]. Mihalko and Krackow [16] reported that the method of osteotomy, as well as the degree of the correction angle, could affect the expected change in leg length according to preoperative planning. In cases requiring a large correction angle to correct a large varus deformity, the leg length is expected to be longer; for example, a 25-degree correction in the lower leg is expected to increase the length from 0.65 cm to 1.05 cm with conventional CWHTO and from 3.35 cm to 3.65 cm with OWHTO [17]. In summary, even with conventional CWHTO-which requires large bony resection of the tibia, as severe varus is corrected to slight valgus alignment-leg length is not always shorter. Our results showed that h-CWHTO was able to maintain leg length, even though the tibial length decreased by a mean of 6.39 mm after surgery; therefore, h-CWHTO is the preferred surgical technique for patients requiring an unchanged leg length due to the relationship between the leg length discrepancy and the contralateral side.

Our results also indicate that the preoperative opening or closing width were not associated with changes in the entire leg length. Bae et al. reported that the change in limb length was significantly correlated with the correction angle on the navigational system among 30 cases of OWHTO [7]. Magnussen et al. [8] also reported a significant correlation between the number of opening sites and the entire limb length among 101 cases of OWHTO (R=0.23, P=0.003). These discrepancies may be due to the sample numbers and three-dimensional changes, such as rotation of the lower limb. It was recently reported that both CWHTO and OWHTO caused rotational changes (external or internal) associated with the correction angle [17]. Regarding the h-CWHTO group, there was no correlation between the closing angle and the entire leg length, as described in cases of conventional CWHTO [7,8].

Leg length discrepancies are associated with musculoskeletal disorders, such as low back pain, OA of the hip, standing balance, and running injuries [18]. The degree of the leg length discrepancy that introduces musculoskeletal disorders is not clearly defined. Gait analyses have shown that a leg length discrepancy of >1 cm results in gait asymmetry [19]; therefore, even though the leg length increased by 6 mm in our study after OWHTO, this leg length discrepancy may have no clinical effect. However, the self-perceived leg length discrepancy was different from the objective leg length discrepancy [20]; thus, patients need to be informed of the possibility of this after OWHTO. h-CWHTO may be a better procedure for patients who do not desire a change in leg length.

Since h-CWHTO is a three-dimensional osteotomy in which the tibia is completely separated and fixed, flexion contracture can be improved by fixing the fragment in the extended position when using a locking plate. Flexion contractures of <30 degrees are thus indicated for surgery [15]. Our results showed that flexion contracture significantly improved from 7.1 degrees to 4.7 degrees in the h-CWHTO group. OWHTO combined with notchplasty was recently reported to improve flexion contracture [21]. In our study, OWHTO did not improve flexion contracture after surgery; this may be because notchplasty was not performed. We believe that the correction on the sagittal plane also had a positive effect on maintaining the leg length in the h-CWHTO group.

Our study has several limitations. First, anteroposterior full-length lower limb radiographs were used to evaluate the lower leg length; there is, therefore, a possibility of radiographic measurement errors due to rotation and knee flexion, both of which may have influenced the measurement results. Nevertheless, when possible, lower limb 3D modeling provided an accurate evaluation, as previously reported [22]. Second, since the indications for OWHTO and h-CWHTO were different, cases with a flexion contracture of >15 degrees were treated using h-CWHTO. Generally, while OWHTO does not improve flexion contracture, it may still affect the results. Third, the h-CWHTO group had more severe varus preoperatively compared to the OWHTO group, which might have influenced the result. Fourth, there were variations in the % MA after surgery. Over- or under-correction may affect lower leg length despite careful preoperative planning and proper surgical techniques; however, postoperative alignment is affected by soft tissue laxity, and some errors must be tolerated [23,24]. Fifth, this study did not include clinical results regarding the leg length discrepancy; therefore, further investigation is needed to clarify the clinical significance of leg length discrepancies after OWHTO and h-CWHTO. Sixth, the sample size in this study was relatively small. Further studies with larger sample sizes or randomized studies are needed to verify the leg length changes after high tibial osteotomy (HTO).

## Conclusions

This study assessed the changes in leg length following open wedge high tibial osteotomy (OWHTO) and hybrid closed wedge high tibial osteotomy (h-CWHTO) techniques. While OWHTO demonstrated extension of the lower leg, h-CWHTO was able to maintain the leg length after surgery. The changes in leg length after OWHTO and h-CWHTO were not associated with preoperative factors. h-CWHTO may be a feasible surgical procedure for patients who do not require leg length changes.
